# Low-Cost Sensor Based on SDR Platforms for TETRA Signals Monitoring

**DOI:** 10.3390/s21093160

**Published:** 2021-05-02

**Authors:** Robert Helbet, Paul Bechet, Vasile Monda, Simona Miclaus, Iulian Bouleanu

**Affiliations:** 1 Department of Electrotechnics and Measurements, Technical University of Cluj-Napoca, 400114 Cluj-Napoca, Romania; vasile.monda@gmail.com; 2 Department of Communications, IT and Cyber Defence, “Nicolae Balcescu” Land Forces Academy of Sibiu, 550170 Sibiu, Romania; pbechet@gmail.com (P.B.); simo.miclaus@gmail.com (S.M.); 3 Department of Computers and Electrical Engineering, “Lucian Blaga” University of Sibiu, 550024 Sibiu, Romania; ibouleanu@gmail.com

**Keywords:** electromagnetic field monitoring, real-time sensor, software defined radio, low-cost, TETRA signals

## Abstract

The paper presents the design and implementation of an electromagnetic field monitoring sensor for the measurement of the Terrestrial Truncked Radio (TETRA) signals using low-cost software defined radio (SDR) platforms. The sensor includes: an SDR platform, a Global Positioning System (GPS) module and a hardware control module. Several SDR platforms having different resolutions of the analog–digital converters were tested in the first phase. The control module was implemented in two variants: a fixed one, using a laptop, and a mobile one, using a Raspberry Pi. The tests demonstrate the following achieved performances: instantaneous acquisition band of 5.12 MHz; dynamic range of the input signal level of (−100 to −30) dBm; frequency resolution of 2.5 kHz; portability and flexibility for use in outdoor environments. The sensor allows complete reporting through amplitude-time-frequency-location descriptors, and in the case of the mobile version, the system performs correctly even at a maximum speed of displacement of 120 km/h. The price of the mobile sensor system variant is approximately EUR 320.

## 1. Introduction

Nowadays, the electromagnetic spectrum has become increasingly crowded, especially due to the development of mobile communications. Thus, it is noticed that channels with different bandwidths are used, from narrowband to wideband. The 4G generation of mobile communications uses variable channel bandwidths, from 1.4 to 20 MHz, while IEEE 802.11n/ac/ax wireless local area networks, to improve data throughput, extend channel bandwidths up to 160 MHz. In addition, channel resource access techniques and digital modulation methods create electromagnetic field variability in both amplitude and time.

The efficient use of resources requires real-time knowledge of the electromagnetic spectrum. In this context, a different approach to monitoring the electromagnetic spectrum is needed. International Telecommunication Union (ITU) has issued several recommendations regarding electromagnetic spectrum monitoring equipment [1]. Thus, due to the large variability in time, it is recommended that the measurements be performed in real time and the analysis of the electromagnetic spectrum include not only an amplitude analysis but also a phase analysis of the spectral components [1]. Monitoring strategies for the electromagnetic spectrum have been reviewed in several countries [2,3,4]. Thus, in the USA, starting in 2013, the National Telecommunications and Information Administration (NTIA) and the National Institute of Standards and Technology (NIST) initiated new electromagnetic spectrum monitoring capabilities [2,5]. By implementing these capabilities, a specific infrastructure for electromagnetic spectrum monitoring has been developed and the results of electromagnetic field measurements can be easily accessed in real time using Internet facilities. To this end, a pilot program involving industry entities, academia and government agencies was developed, with the objective of generating a database called the Measured Spectrum Occupancy Database (MSOD).

Some recent studies have shown that electromagnetic spectrum monitoring suffers from two essential limitations [6,7]: lack or reduced scalability and lack of supporting applications to be operated directly by the monitoring sensor. The electromagnetic monitoring is the responsibility of government agencies, which usually use expensive monitoring stations. This makes scalability limited or even nonexistent. The monitoring stations are usually composed of high-performance spectrum analyzers that already have various functions implemented. This creates a relatively closed environment for further processing of the recorded data.

A low-cost alternative to deploying a spectrum monitoring station was recently offered by Software Defined Radio (SDR) technology. The requirements for large-scale broadband real-time monitoring are successfully met [7]. Due to the relatively low price of SDR platforms, the number of sensors in the entire architecture of the monitoring network may become much higher. Additionally, their small size and flexibility offer the possibility of placing them in different locations. In this way, the qualitative and quantitative descriptors of the electromagnetic environment are substantially improved.

Several electromagnetic spectrum monitoring solutions have recently been proposed—for example, the Microsoft Spectrum Observatories (https://www.google.com/get/spectrumdatabase/ (accessed on 24 March 2021)). A recent project is the ElectroSense project (https://electrosense.org/ (accessed on 24 March 2021)), which is the first initiative that aims to exploit the facilities offered by low-cost programmable spectrum sensors, such as SDR sensors [8,9,10].

The adequacy of SDR platform parameters for real-time and accurate monitoring is an important factor in the design of the monitoring station. This depends on the characteristics of the monitored signals (bandwidth, frequency range, duration of symbols used for data packaging, transmission technique to access the channel resources, spatial distribution of data using Multiple Input-Multiple Output (MIMO) and beamforming techniques, etc.). Recently, specialists have reported several electromagnetic spectrum monitoring stations based on SDR technology [11,12,13,14,15]. The main characteristics of an SDR platform that will influence the performance of a monitoring station are as follows: frequency range, sampling frequency and resolution of the digital–analog converter [16,17,18]. The applications of SDR platforms have expanded as a result of the benefits offered by software processing, a series of results that have been recently reported in many areas [19,20,21,22].

The key factors that define the figure of merit of a sensor for electromagnetic spectrum monitoring are [7]: low cost; small form-factor; wide-band; distributed; real time; commercial off-the-shelf (COTS) hardware; flexibility. The SDR receiver proposed in [23] has a recording time of 4 s, and the power spectrum is computed via Fast Fourier Transform (FFT), at a resolution of 8192 points. For a sampling frequency of 100 MHz, a frequency resolution of 100 MHz/8192 = 12.207 kHz resulted. Other features are the following: frequency range of (9 kHz–3500 MHz); the noise factor of the receiver is 6 dB; the resolution of the digital analog converter is 16 bits; search speed is 1 GHz/s and real-time acquisition band is at least 16 MHz [23].

A sensor whose structure is based on low-cost commercial off-the-shelf (COTS) hardware components is proposed in [7]. The radio interface is the RTL-SDR USB platform and allows a typical sampling frequency of 2.4 MS/s. The cost of such a sensor is less than USD 100. Comparative evaluations of a Universal Software Radio Peripheral (USRP) SDR platform have shown that the sensitivity is approximately 10 dB lower than the USRP’s. The low-cost monitoring system is able to sense, analyze and compress a lossless 2.4 MHz band at a frequency resolution of less than 10 kHz [7].

Another electromagnetic field sensor that also has a radio interface of the RTL-SDR USB platform is presented in [8]. The sensor is configured in two acquisition modes. The first mode allows determination of the power spectral density (PSD) by implementing processing operations at the sensor level (sampling windowing, FFT, averaging, data compression). The second acquisition mode does not use processing at the sensor level, but directly records the In-phase and Quadrature (IQ) components of the raw signal samples. The RTL-SDR radio interface is controlled by a Raspberry Pi device to reduce the cost. Depending on the compression factors and data averaging, transfer rates of the order of 50–100 Kb/s are obtained in the PSD mode, while much higher data transfer rates are obtained, up to the 50 Mb/s, in the IQ acquisition mode [8].

The present paper aims to design and implement an electromagnetic spectrum monitoring station with real-time analysis capabilities to track signals emitted in the TETRA communications standard. The TETRA standard was designed for emergency situations and is used primarily by government agencies [24,25].

A sensor for monitoring TETRA signals was initially tested by the authors of this paper and the preliminary results are reported in [26]. This sensor has been improved and thus new TETRA signal monitoring capabilities have been developed.

The main contributions of the current work are:

1. *Integration of a Geo-Location module and new support applications have been added*
  A low-cost Global Navigation Satellite System (GNSS) module has been integrated into the sensor. A software application has been added to calculate the power of each TETRA channel. The readings from the GNSS module were synchronized with the measurement of the power of the TETRA channels.
2. *Real-time processing capabilities and instantaneous acquisition frequency bandwidth have been improved*
  An extension has been added through which the sensor can be used either for the Uplink frequency range or for the Downlink frequency range. In this way, it is possible to monitor the emissions of both the terminal and the base stations. Several SDR platforms on the market have been tested. A platform has been chosen and integrated into the monitoring sensor that allows real-time acquisition of the full range of TETRA signals.
3. *COTS sensor and a small form-factor have been achieved*
  In order to improve the portability of the sensor its size and weight have been reduced substantially. The design and implementation of the sensor took into account the choice of COTS hardware components. The SDR platform was controlled with a Raspberry Pi device. This led to the low price of the sensor.

The main benefit of our sensor is that the data are collected simultaneously, in real time, for all TETRA channels, and the information, after processing, can be obtained in detail for each TETRA channel separately. As a general goal, we aimed to achieve a sensor capable of providing a complete real-time characterization of TETRA signals in all four dimensions: amplitude, time, frequency, space (location).

Next, the work is organized as follows: in Section 2, the measurement sensor based on the SDR platform is presented in detail and the method of monitoring the electromagnetic spectrum specific to the TETRA standard is explained. Section 3 presents the results following the calibration of the system in laboratory conditions and testing in real environmental conditions. Section 4 formulates the general conclusions of the work.

## 2. Material and Methods

The main features of the TETRA standard are [24]: 25 kHz channel bandwidth; 200 channels in a 5 MHz frequency band; duplex working mode (between 380 and 385 MHz for mobile station (MS) and between 390 and 395 MHz for base station (BS)); access to communication channel resources based on frequency access techniques (Frequency Division Multiplexing Access—FDMA) but also in time (Time Division Multiplexing Access—TDMA). From the perspective of real-time acquisition, the following characteristics must be taken into account: frame length: 56.67 ms; slot duration: 14.167 ms; duration of a multiframe (18 frames): 1.02 s. According to the specifications of the TETRA standard, a monitoring station devoted to BS measurement should simultaneously acquire 200 channels, each with a bandwidth of 25 kHz, in the frequency range from 390 to 395 MHz, with an acquisition time of less than 14 ms.

### 2.1. The Procedure of TETRA Signal Channel Power Determination

**Figure 1:**
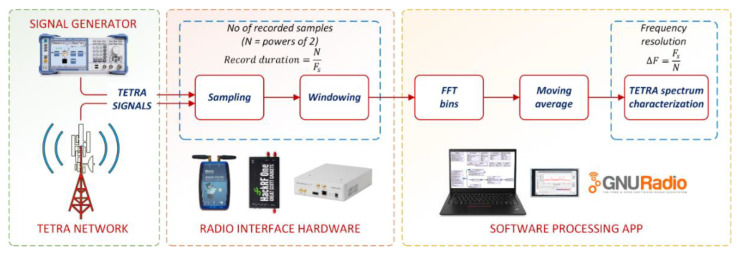
Data acquisition and processing steps used to characterize, in detail, the TETRA signals.

Figure 1 shows the processing operations through which the monitoring results of the TETRA signals were obtained. In the case of measurements made in laboratory conditions, TETRA signals are provided by a signal generator, while in the case of measurements in real conditions, they were obtained by means of a receiving antenna scanning the TETRA network emissions.

The radio interface based on the SDR platform acquires the TETRA signals, transforms them into digital format and records them sequentially. Subsequently, the IQ signal samples obtained at the output of the radio interface are applied to the FFT processor for characterization in the frequency domain. The frequency resolution obtained is given by the ratio between the sampling frequency Fs and the number of points N necessary for the FFT calculation, as shown in Relation (1), based on [27]:

(1) $$\Delta F=\frac{F_{s}}{N}$$

If the sampling frequency $F_{s}$ is 5.12 MHz and the number of points *N* is 2048, a resolution of 2.5 kHz is obtained. If we take into account the fact that the bandwidth of the TETRA channel is 25 kHz, it means that 10 points are used for the calculation of the channel power. Through software applications, following the processing of spectral lines resulting from the application of FFT, the TETRA signals can be characterized in detail (for example, the power of each channel can be calculated, etc.).

**Figure 2:**
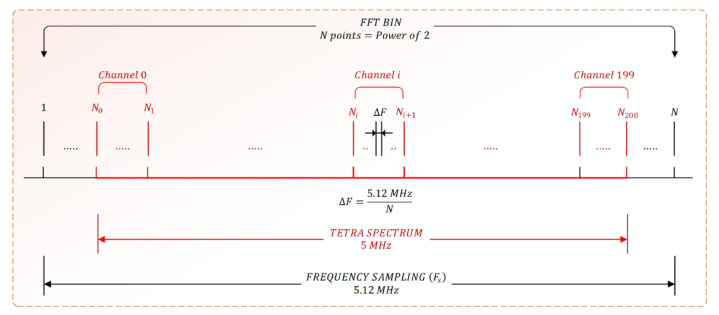
Correspondence between the spectral lines obtained after applying FFT and the TETRA channels.

The method of the TETRA channel power calculation is presented graphically in Figure 2. At each FFT bin, the details of the TETRA signals will be considered. Starting from the fact that the TETRA spectrum occupies a bandwidth of 5 MHz centered on the frequency of 382.5 MHz in the case of Uplink and on 392.5 MHz in the case of Downlink, a sampling frequency of 5.12 MHz was established for the FFT calculation. In this way, a bandwidth of 0.12 MHz (0.06 MHz left and right, respectively, of the TETRA spectrum) remains outside of the spectrum of interest. The power of a TETRA channel is calculated through the amplitudes of the spectral lines inside the channel. Thus, for this purpose, the mathematical expression presented in [28] will be applied (2):

(2) Pchi=10⋅lg(BchBNIF⋅1Ni+1−Ni⋅∑NiNi+110Pi10)

where: *P_chi_* represents the power expressed in dBm calculated for channel *i* (*i* = 0, …, 199); *B_ch_* represents the TETRA channel bandwidth (in this case 25 kHz); *B_NIF_* represents the band of the noise filter used; *P_i_* represents the power in dBm corresponding to each pixel (spectral line); *N_i+1_* and *N_i_* represent the indexes of the measurement points for each TETRA channel.

In general, the points between which we find the channel *i* (*i* = 0, …, 199) are determined with the relations of (3):

(3) $$\left\{ \begin{array}{l} N_{i}=i\cdot \frac{0.025}{\Delta F}+\frac{0.06}{\Delta F}+1;\;\;\;\;i=0,\ldots ,199\\ N_{i}{}_{+1}=(i+1)\cdot \frac{0.025}{\Delta F}+\frac{0.06}{\Delta F}+1;\;i=0,\ldots ,199\\ \mathrm{Frequency}\;\mathrm{Resolution}=\Delta F[\mathrm{MHz}]=\frac{F_{S}[\mathrm{MHz}]}{N} \end{array}\right.$$

Therefore, the selection of a TETRA channel is made by identifying the spectral lines corresponding to the channel and the calculation of the channel power takes place further by applying Relation (2).

### 2.2. Sensor Design and Implementation: Software Applications

The software applications were designed and implemented in the free and open-source GNU Radio software development toolkit. In 2001, Eric Blossom began implementing the GNU Radio project in order to provide a general framework for SDR platforms in terms of software control [29].

Software application for data control and processing in the case of our sensor includes several modules. Some of them are made with existing signal processing blocks available in libraries of the GNU Radio toolkit, others have been written and implemented especially for our sensor needs.

**Figure 3:**
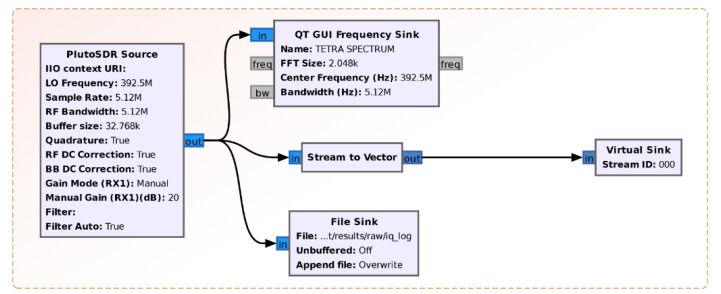
Radio interface module to SDR platform and data saving in IQ format.

Figure 3 shows the radio interface module of the SDR platform. The module is built with existing GNU Radio blocks and it also includes: a block for displaying the spectrum (QT Frequency Sink), a block for saving IQ samples (File Sink) and a block that prepares the *N* samples for the purpose of FFT calculation (Stream to Vector). The vector size is given by the number of FFT calculation points (FFT_size).

**Figure 4:**
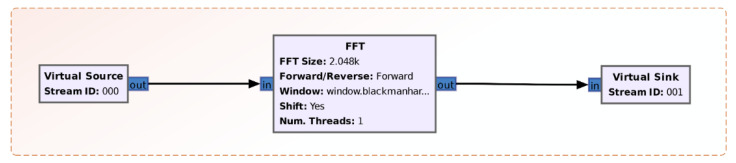
FFT calculation method in GNU Radio.

In Figure 4, the module of data logging and FFT calculation is presented. The block can be found in the GNU Radio libraries. The number of calculation points of the FFT is imposed by means of the variable FFT_size. Additionally, depending on the purpose, different types of windows can be chosen: rectangular, hamming, blackman, etc.

**Figure 5:**
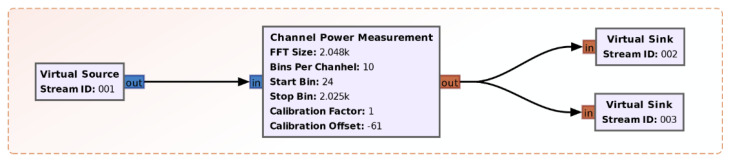
The TETRA channel power computation module.

Figure 5 emphasizes the power calculation module of the TETRA channel (Channel Power Measurement). The module was written specifically for this purpose in Python and imported into GNU Radio. Basically, this module implements the procedure presented in Section 2.2.

**Figure 6:**
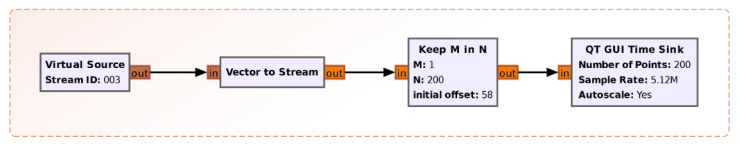
TETRA channel selection and the channel power display module.

In Figure 6, we show the module devoted to the selection the TETRA channel and to the displaying of its power. The module consists of the GNU Radio blocks already available.

**Figure 7:**
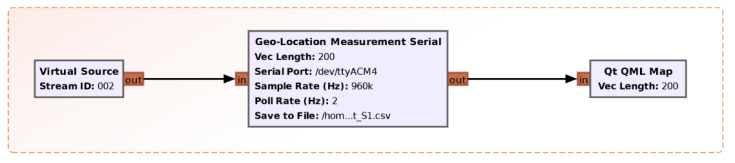
The Geo-Location module.

Figure 7 shows the Geo-Location module. This module was written specifically in Python and has the role of integrating the measurements provided by a low-cost GNSS receiver into our monitoring sensor. The reason for choosing the GNSS receiver was that it has the advantage of accessing several satellites simultaneously. This leads to an increased positioning accuracy, a decreased redundancy and an increased availability of the received positioning data, even when the field of view of satellites is partially obstructed.

The used GNSS receiver is able to catch signals from the GPS and GLONASS satellite constellations and manages to have a positioning accuracy of about 2.5 m. Data from the GNSS receiver are transmitted to the sensor using the NMEA-0183 protocol. The Python application interprets the Recommended Minimum Specific GNSS Data (RMC) messages and synchronizes the position with the measurements made on the TETRA signals. The data are then saved in a *.csv file type for further processing. Additionally, through the Qt QML Map block, the data are displayed on the map.

### 2.3. Sensor Testing Methodology in the Laboratory

**Figure 8:**
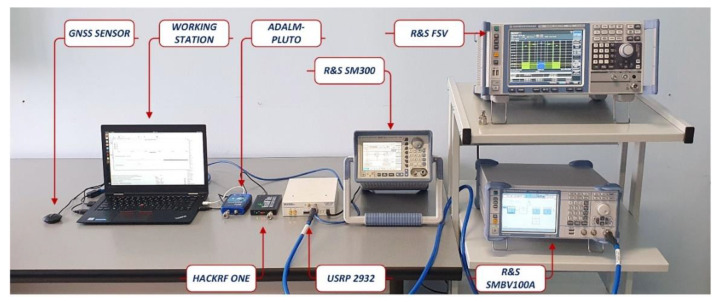
Measurement set-up for laboratory testing.

The TETRA signals’ monitoring sensor was tested in laboratory conditions based on three SDR platform models: ADALM Pluto, Hack RF One and NI USRP 2930. The results of the measurements performed with our sensor were compared with those performed by a high-performance Rohde & Schwarz spectrum analyzer, model FSV13, which was used as reference. The TETRA signal source was provided by a Rohde & Schwarz SMBV 100 A vector signal generator. Additionally, in the sweep mode, the Rohde & Schwarz SM 300 vector signal generator was used. Figure 8 shows the measurement set-up containing the three SDR platform models, the R&S SMV 100 A signal generators, the R&S SM300 and the R&S FSV13 spectrum analyzer.

Three SDR platform models were chosen based on the following criteria: low-cost price (for this purpose, the SDR platforms ADALM Pluto and Hack RF One were chosen); real-time monitoring capabilities for a bandwidth of at least 5 MHz (for this purpose, the minimum acquisition band for each platform must be 5 MHz); small dimensions and the possibility of using a USB power supply in order to have a portable sensor (this is accomplished by the low cost of ADALM Pluto and Hack RF One platforms); high-quality monitoring capabilities (sensitivity, amplitude dynamic range, etc.) if precise measurement results are expected (for this purpose, the SDR NI USRP 2930 platform was chosen).

The HackRF One platform has the following characteristics: frequency range: 1 MHz to 6 GHz; instantaneous bandwidth: up to 20 MHz; interface: USB 2.0; analog digital converter (ADC) resolution: 8 bits. The ADALM Pluto platform has the following characteristics: frequency range: 325 MHz to 3.8 GHz; up to 20 MHz of instantaneous bandwidth; 12-bit ADC resolution. The two platforms are low-cost and were chosen from the perspective of analyzing the influence of the resolution of the digital analog converter on the sensor performance. For the NI USRP 2930 platform: frequency range: 50 MHz to 2.2 GHz; 14-bit ADC resolution; maximum real-time instantaneous bandwidth: 20 MHz; noise figure: 5 to 7 dB.

The laboratory tests of the sensor included: the frequency response in the range of TETRA signals (for Downlink, between 390 and 395 MHz); analysis of the amplitude dynamic range; adjacent channel interference attenuation analysis; determining the sensor’s own noise level; analysis of the influence of the number of FFT points on the sensor’s performance. The tests comprised all three SDR platforms, and a comparison was made with the reference values obtained by using the professional R&S FSV13 analyzer in the same measurement conditions.

The R&S SM300 signal generator was used to study the frequency response. Its settings were: sweep operating mode in the frequency range from 390 to 395 MHz; frequency step: 1 kHz; dwell time: 200 ms.

The study of the dynamic amplitude range was performed using the R&S SMBV 100 A generator as a signal source emitting a TETRA channel (channel 131, central frequency 393.2875 MHz) with an output level from −112 to −32 dBm.

The attenuation of the adjacent channel was determined both for the 1st order adjacent channels and for the 2nd order adjacent channels. For this purpose, an R&S SMBV 100 A signal generator was used.

The study of the noise of the SDR platforms and of the R&S analyzer FSVR13 was performed by measuring the noise power of the TETRA channel in the conditions in which the load impedance of 50 ohms was connected to the RF input.

An analysis of the influence of the FFT number of points was performed taking into consideration three cases: 2048 points (frequency resolution of 2.5 kHz); 4096 points (frequency resolution of 1.25 kHz) and 8192 points (frequency resolution of 0.625 kHz).

### 2.4. Sensor Testing Methodology in the Real Environment

The design and implementation of the sensor were based of the assumption of achieving a low-cost sensor with a high degree of portability, to be simply used for outdoor measurements. The size, weight and power are important requirements. The sensor’s control was achieved in two variants: (a) with a laptop; (b) with a Raspberry Pi device. The small single-board computer had the following characteristics: Processor: Broadcom BCM2711, Quad core Cortex-A72 (ARM v8) 64-bit SoC @ 1.5 GHz; RAM: 4 GB LPDDR4-3200 SDRAM; Connectivity: 2.4 GHz and 5.0 GHz IEEE 802.11ac wireless, Bluetooth 5.0, BLE, Gigabit Ethernet; Power supply: 5 V DC via USB-C connector (minimum 3 A).

**Figure 9:**
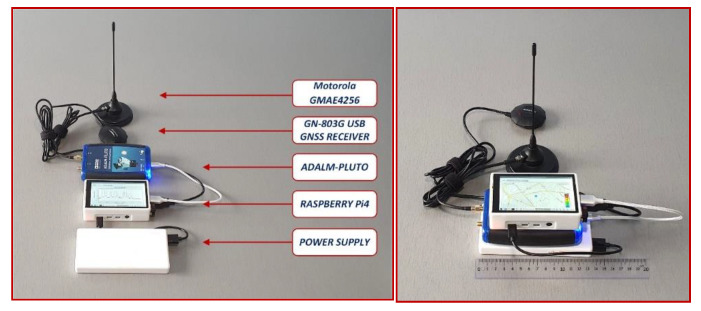
Portable sensor elements controlled with the Raspberry Pi.

To meet the mobility conditions and to ensure the local control interface of the Raspberry computer, it was equipped with a high-performance display model HyperPixel 4.0. The protection of the components of the Raspberry device was provided by preparing a 3D printed resin housing (short curing time, high level of details with firm edges and smooth surfaces). The housing has a system of holes that allow optimal operation of the fan by passing the air flow to the outside. Figure 9 shows the portable sensor with all its components. The approximate cost of such a sensor is EUR 320 (ADALM Pluto SDR platform: approx. EUR 130; HiperPixel 4.0 touch display: approx. EUR 40; Raspberry Pi: approx. EUR 50; Cooling fan: approx. EUR 10; Housing: approx. EUR 10; Motorola GMAE4256 magnetic antenna: EUR 45; GN-803G USB GNSS GPS GLONASS Receiver: EUR 12; External accumulator Huawei SuperCharge, rated capacity 6800 mAh-5 V 4 A: EUR 20).

**Figure 10:**
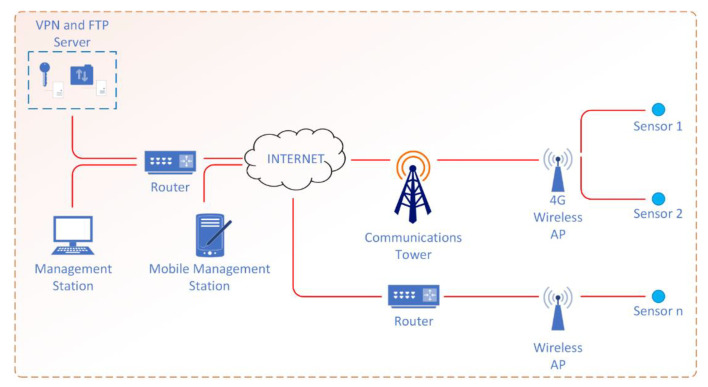
Network architecture for the remote management of the sensors.

A virtual private network (VPN) was implemented for remote sensor management. The network architecture is shown in Figure 10. The VPN server was deployed on a Raspberry Pi 3B+ computer, using the open source OpenVPN solution. The sensors were configured to automatically connect to the VPN when they start, when there is an Internet connection available. This creates a private virtual network that works regardless of how customers (sensors) are connect to the Internet. The system administrator thus has direct access to the sensors regardless of how they connect to the Internet. They can establish Secure Socket Shell (SSH) connections to configure the operating system, or can view and operate directly in the graphical interface of the sensors using the graphical desktop interface sharing system, Virtual Network Computing (VNC).

The Internet connection of the sensors is made through Wi-Fi access points, in the 2.4 or 5 GHz bands, with the communication infrastructure being provided by both mobile data service providers and data service providers through wired networks (in the case of sensors located in fixed locations). The files resulting from the measurements and deposited in the storage memory of each sensor are automatically synchronized on the File Transfer Protocol (FTP) server, at programmable time intervals, using the rsync utility. This has the advantage of greatly reducing data traffic in the network as the synchronization of files is carried out by checking the time of files’ modifications and their sizes, with the data transmission taking place only if necessary.

In order to process the data recorded by the field sensors, a software application has been written. It allows different analyses—for example, the representation of the electromagnetic field level measured by a particular sensor on the map, on any of the 200 TETRA channels.

The field testing (in situ) of the sensor took into account not only the static sensor, but also the moving one. Therefore, its placement on a vehicle was considered, while data collection through the private remote management network and the subsequent analysis of the results were enabled. The itineraries of the vehicle carrying the sensor were chosen so as to allow a constant travel speed. A study of the influence of certain different dynamics situations on data accuracy of the TETRA channels measurements was also performed.

## 3. Results and Discussion

### 3.1. Results of the Laboratory Testing

**Figure 11:**
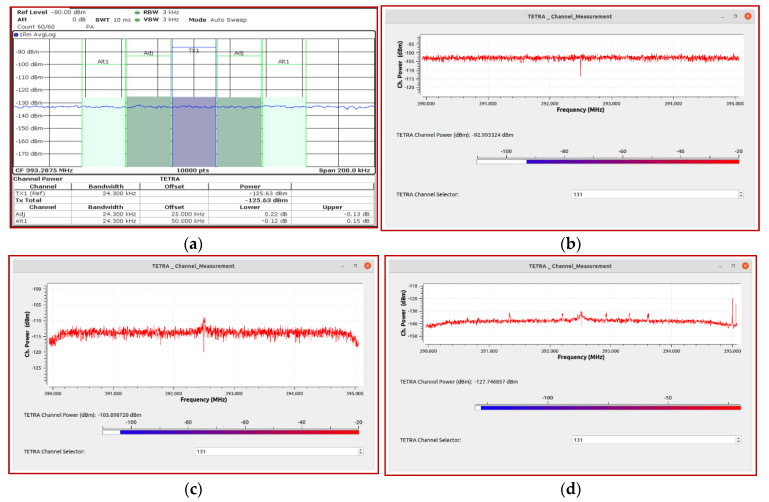
Display average noise level in the frequency band (390–395) MHz: (**a**) R&S FSV13; (**b**) HackRF One; (**c**) ADALM Pluto; (**d**) NI USRP 2930.

Noise power levels in the TETRA channel for the three types of SDR platforms and for the R&S FSV13 spectrum analyzer used as a reference are presented in Table 1 (the measurements were performed under the conditions of connecting an impedance of 50 ohms to the RF inputs). The number of FFT points was 2048 and the resolution bandwidth (RBW) of the spectrum analyzer was 3 kHz. In Figure 11, the noise floor can be observed for the entire TETRA Downlink band, between 390 and 395 MHz. In order to accurately measure the signal strength of the TETRA channel, we determined that a signal-to-noise (SNR) ratio of at least 5 dB is required. This means that the minimum power level that can be measured with the the SDR platforms is: −119.6 dBm for NI USRP 2930; −98.2 dBm for ADALM Pluto; −85.8 dBm for Hack RF One.

The R&S SMBV 100 A generator set on a TETRA channel (channel 131, center frequency 393.2875 MHz) was used as a signal source to study the dynamic range of the amplitude. Its output level can vary between −112 dBm and −32 dBm. The measurement results are gathered in Table 2. The power levels displayed by the SDR NI USRP 2930 platform are very similar to those provided by the R&S FSV13 spectrum analyzer, considered as a reference. The ADALM Pluto SDR platform has a good response for an amplitude range of the input signal between −97 dBm and −32 dBm, while the SDR Hack RF One platform represents well only the amplitude range between −82 dBm and −32 dBm. These values represent the limits established by the different sensitivities of the two SDR platforms.

**Figure 12:**
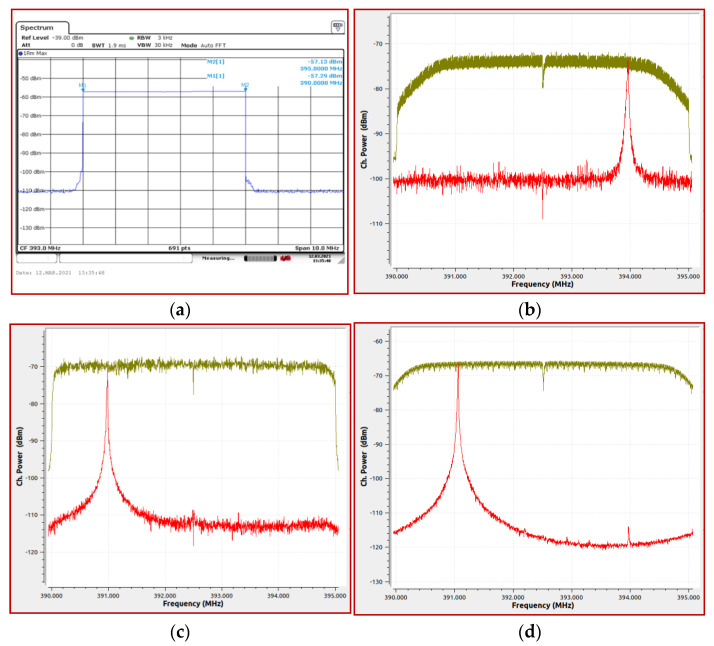
The frequency response, comparatively, in the frequency range (390–395) MHz: (**a**) R&S FSV13; (**b**) HackRF One; (**c**) ADALM Pluto; (**d**) NI USRP 2930.

Figure 12 provides a comparative observation of the frequency response in the range of interest. The signal was emitted by the R&S SM300 signal generator having the following settings: sweep operating mode in the frequency range (390–395) MHz; frequency step: 1 kHz; dwell time: 200 ms. It can be seen that, by comparison with the R&S FSV13 spectrum analyzer’s response, the best behavior is provided by NI USRP 2930 platform, followed by the SDR ADALM Pluto platform. This result is explained by the good resolutions of the digital analog converter of the two platforms (12 bits for ADALM Pluto and 14 bits for Ni USRP 2930, respectively).

**Figure 13:**
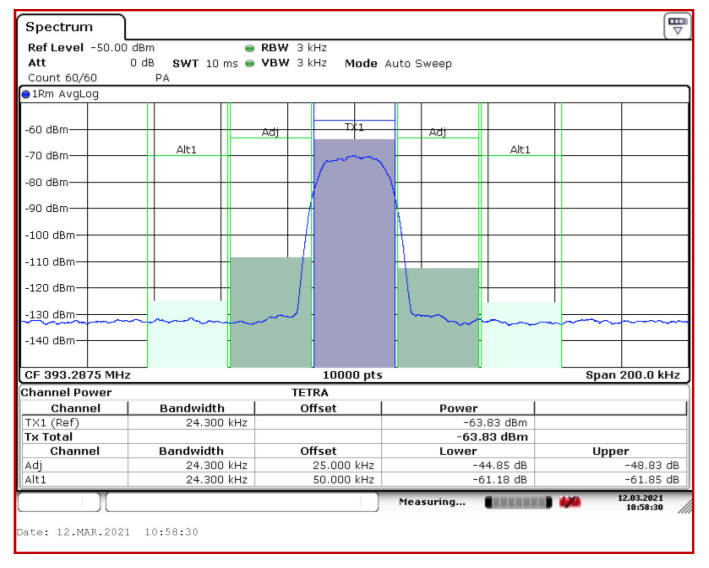
Adjacent channel attenuation measured by R&S FSV13 analyzer.

The sensor has been designed to be able to acquire all 200 TETRA channels simultaneously, therefore an important parameter is the adjacent channel interference (ACI). Figure 13 shows the levels of the adjacent channels measured with the R&S FSV13 analyzer in the situation when a TETRA signal was applied to the input, respectively channel number 131, with a level of −62 dBm. A comparison was made with the results provided by the other three SDR platforms. A synthetic view of attenuation of the adjacent channels is given in Table 3.

In the case of SDR platforms, a resolution of 2048 points was used for the FFT calculation. Even in this situation, the results obtained with the SDR NI USP 2930 platform are comparable to those provided by the R&S FSV13 reference analyzer. The ADALM Pluto SDR platform has an attenuation of approximately 30 dB for adjacent level 1 channels and approximately 35 dB for adjacent level 2 channels. These attenuations may be insufficient in the case of field measurements, especially in the case of strong channels in the immediate vicinity of the TETRA emission sources. For example, a TETRA channel with a power of −40 dBm will produce adjacent channel levels of −60 dBm when measured with the ADALM Pluto SDR platform. This quantity can lead to misinterpretations regarding the presence of TETRA signals in different frequency bands.

A solution of increasing the attenuation of the adjacent channel consists in improving the frequency resolution, by increasing the number of FFT points of calculation. Table 4 compares the attenuations of the adjacent channel for measurements made with the SDR ADALM Pluto platform for the values of the frequency resolution of: 2048 points, 4096 points and 8192 points, respectively. We observe that the attenuation of the adjacent channels for a resolution of 8192 points is about 60 dB. This size can be considered sufficient, even in the case of strong TETRA channels. For example, for a TETRA channel with a power of −40 dBm, an adjacent channel level of less than −100 dBm will result.

### 3.2. Results of the Field Measurements (Real Case)

**Figure 14:**
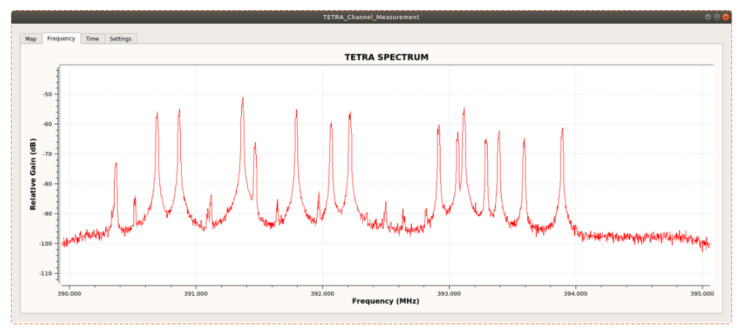
Downlink spectrum of TETRA channels measured in a field location by the ADALM Pluto SDR platform.

Figure 14 shows the Downlink spectrum of the TETRA signals following the acquisition made with the portable sensor in the field (SDR ADALM Pluto platform). Several active channels of different amplitudes can be seen. Additionally, 10 dB higher levels than the floor noise are observed, even for the channels with the lowest levels.

**Figure 15:**
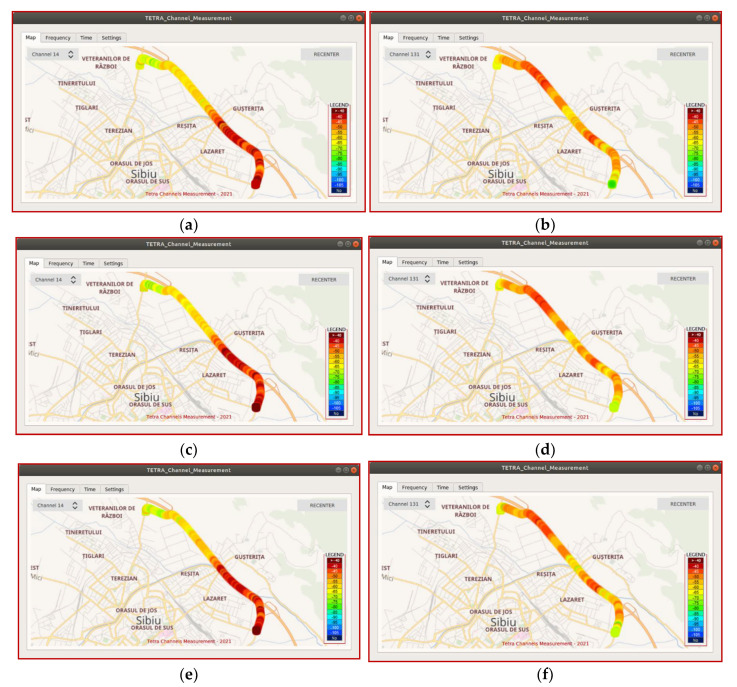
Representation of the measured TETRA channel powers (channel no. 14 and no. 131) when the receiving sensor was moving constantly on a route at different speeds: (**a**) channel 14, speed—60 km/h; (**b**) channel 131, speed—60 km/h; (**c**) channel 14, speed—90 km/h; (**d**) channel 131, speed—90 km/h; (**e**) channel 14, speed—120 km/h; (**f**) channel 131, speed—120 km/h.

For testing the sensor and the GNU Radio field application in motion, the sensor was placed inside a vehicle. Three measurements were made successively in which the vehicle moved with constant but different speeds: 60, 90, and 120 km/h, respectively. Figure 15 comparatively presents the power levels measured on TETRA channel no. 14 (center frequency 390.3625 MHz) and no. 131 (center frequency 393.2875 MHz). It can be visually observed that, on the route, the displayed power levels for the two TETRA channels are comparable, regardless of the value of the vehicle speed.

**Figure 16:**
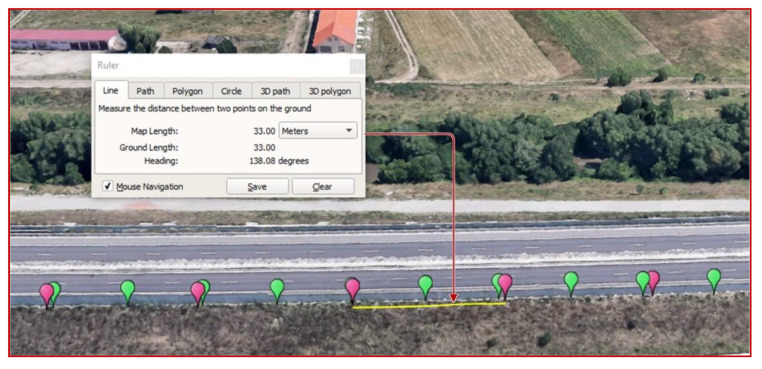
Evaluating the distance between two consecutive measurement points (Google Earth) for space resolution assessment in the case of the moving sensor.

In terms of the accuracy of the positioning of the measuring points by the GNSS receiver, in Figure 16 we can observe the distance between two consecutive points measured with the Google Earth application. In the case of 120 km/h speed, compared to the theoretical distance of 33.33 m between two consecutive points, it was observed that the measured distance is 33 m. The difference of 0.33 m is insignificant and falls within the positioning accuracy of the GNSS receiver.

**Figure 17:**
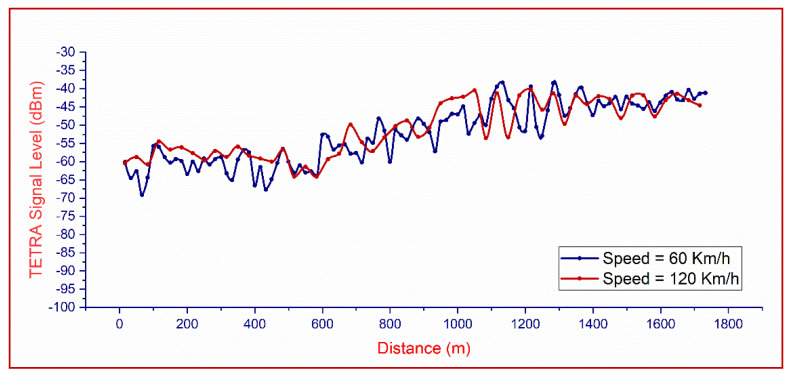
TETRA channel measured power with the mobile sensor: the speed is not influencing the correctness of the readings—exemplified by the speeds of 60 and 120 km/h.

Figure 17 indicates the measured power levels of a TETRA channel, when the sensor is moving with constant speeds of 60 and 120 km/h, respectively, along a short section extracted from the monitoring itinerary. We observed that the influence of the sensor’s speed on the measurement results is insignificant, even when doubling the speed. This illustrates that placing a sensor for monitoring TETRA signals on a mobile tracker, even if it will have a variable speed, does not lead to misinterpretations of the characteristics of the acquired signals (position, amplitude, etc.).

### 3.3. Discussion

The sensor test results show the following characteristics: real-time acquisition bandwidth of 5.12 MHz; sensitivity of −100 dBm for the handheld version and −120 dBm for the fixed version; dynamic range of the input signal: −100 to −30 dBm; frequency resolution better than 2.5 kHz; sensor made up of COTS hardware components; small form-factor: 13.5 (width) × 7 (height) × 7 cm (depth); weight (cables included): 529 g; weight (no cables): 506 g; weight (no battery): 280 g. The approximate cost of the sensor is EUR 320.

Compared to other SDR sensors reported recently [6,7,8], our sensor brings the following contributions:

1. *Improved real-time acquisition bandwidth*
  Our sensor has been tested under conditions of an instantaneous acquisition bandwidth of 5.12 MHz. Depending on the characteristics of the signals to be monitored, we can extend this instantaneous bandwidth up to 20 MHz.
2. *Good amplitude frequency response and improved sensor sensitivity*
  The selection of SDR platforms with good resolution for analogue-to-digital conversion (12 bits for the portable version of the sensor and 14 bits for the fixed version) improves sensitivity as well as dynamic range. Compared to 8-bit SDR platforms such as those reported in [6,8], we estimate an improvement of at least 10 dB in sensitivity terms.
3. *Improved data storage and data transfer rate with the addition of supporting applications*
  Direct storage of IQ data can lead to a large amount of data. This can limit both data transfer and real-time data processing. From this point of view, it is useful to implement support applications that are directly operated by the sensor. In [8], it is shown that the acquisition of IQ samples with a bandwidth of 2.4 MHz generates a data transfer rate of about 50 Mb/s. Adding a support application to the sensor for power spectral density (PSD) computation, after data averaging and data compression, reduces the throughput to 50–100 kb/s. In the case of our sensor, the support application for the TETRA channel power computation directly by the sensor further reduces the data transfer rate. A rate of 15 kb/s is obtained even if the acquisition bandwidth is 5.12 MHz and the SDR platform resolution is 12 bits.

To our knowledge, this is the first SDR sensor for real-time monitoring of TETRA signals. The sensor can be easily accommodated in any vehicle without imposing additional requirements (space, power supply, etc.). Sensor portability, autonomy in operation and low price offer the possibility to increase the number of sensors. A complete characterization of the TETRA spectrum becomes possible in four dimensions: amplitude, frequency, space and time.

## 4. Conclusions

The present paper aimed to design, implement and test a sensor based on SDR technology that is able to monitor the entire electromagnetic spectrum of the TETRA signals.

We focused specifically on achieving a sensor that would allow large-scale monitoring of the electromagnetic spectrum. We obtained a sensor made with COTS hardware components and with technical characteristics comparable to those of a high-performance monitoring system built by well-known suppliers. The advantage of this is that the cost is very low.

The sensor has been tested both in laboratory conditions and in real outdoor operating conditions. Real-time information on all TETRA channels can solve problems such as: radio coverage determination, signal classification based on the values of channel power; intrusion or interference detection. The accuracy of the sensor was validated by comparing the measurements with a high-performance spectrum analyzer. The evaluation of the sensor will continue with the implementation of an uncertainty budget calculation procedure for the measurements. For this purpose, the combined standard uncertainty for the measured power levels will be determined by extracting probability density functions of the uncertainties of the input quantities and then applying the uncertainty equation.

Future research directions consist of: expanding storage capacities at the level of the monitoring network by creating a database, especially with IQ signal samples; testing of Big Data and Machine Learning algorithms in order to process the data resulting from the long-term acquisition; expanding the monitoring capabilities of low-cost sensors and other signals, especially in the case of broadband signals (for which the instantaneous acquisition must cover bandwidths of the order of 100 MHz).

## Figures and Tables

**Table 1:** Noise power level in a TETRA channel (channel 131, central frequency 393.2875 MHz).

R&S FSV13	NI USRP 2930	ADALM Pluto	Hack RF One
−125.6 dBm	−124.6 dBm	−103.2 dBm	−90.8 dBm

**Table 2:** The dynamic range of the amplitude for an emitted TETRA channel signal varying between −112 dBm and −32 dBm (channel no. 131, central frequency 393.2875 MHz).

Output Power Signal Generator SMBV 100 A [dBm]	Channel Power FSV13 [dBm]	Channel Power NI USRP 2930 [dBm]	Channel Power ADALM Pluto [dBm]	Channel Power Hack RF One [dBm]
−32	−33.2	−34.3	−33.2	−33.3
−42	−43.6	−43.5	−43.3	−43.3
−52	−53.5	−53.4	−53.2	−53.3
−62	−63.6	−63.5	−63.2	−63.2
−72	−73.5	−73.5	−73.2	−74.0
−82	−83.4	−83.4	−83.1	−83.3
−92	−93.4	−93.2	−92.7	-
−97	−98.6	−97.9	−97.6	-
−102	−103.4	−102.7	-	-
−107	−108.2	−108.8	-	-
−112	−113.5	−113.2	-	-

**Table 3:** Attenuation of adjacent channels by the three types of SDR platforms and for the R&S FSV13 spectrum analyzer (TETRA channel no. 131, input level −62 dBm).

Equipment	Adj. Ch. Level 1 Lower [dB]	Adj. Ch. Level 1 Upper [dB]	Adj. Ch. Level 2 Lower [dB]	Adj. Ch. Level 2 Upper [dB]
R&S FSV13	−44.85	−48.83	−61.18	−61.85
NI USRP 2930	−42.2	−46.3	−60.5	−60.28
ADALM Pluto	−29.9	−27.6	−35.63	−34.2
Hack RF One	−25.3	−26.3	−27.3	−28.2

**Table 4:** Influence of FFT resolution on the attenuation of the adjacent channels, for the ADALM Pluto SDR platform (TETRA channel no.131, input signal level −42 dBm).

FFT Number of Points	Adj. Ch. Level 1 Lower [dB]	Adj. Ch. Level 1 Upper [dB]	Adj. Ch. Level 2 Lower [dB]	Adj. Ch. Level 2 Upper [dB]
2048	−30.6	−27.8	−36.8	−35.5
4096	−44.1	−42.5	−49.9	−49.2
8192	−59.2	−58.7	−59.7	−59.4
